# Duodenal Varices: A Case Report and Review of the Literature

**DOI:** 10.1155/1995/97496

**Published:** 1995

**Authors:** Lawrence McChesney, Donald Jensen, Terence Matalon, Daniel Ganger, Howard Sankary, Preston Foster, James W. Williams

**Affiliations:** 1 Transplantation Surgery Rush University Rush- Presbyterian-St. Luke's Medical Center Chicago Illinois 60612 USA; 2 Clninical Hepatology Rush University Rush- Presbyterian-St. Luke's Medical Center Chicago Illinois 60612 USA; 3 Invasive Radiology Rush University Rush- Presbyterian-St. Luke's Medical Center Chicago Illinois 60612 USA


*HPB Surgery*, 1995, Vol. 9, pp. 31–35
Reprints available directly from the publisher
Photocopying permitted by license only

© 1995 OPA (Overseas Publishers Association)
Amsterdam B.V. Published in The Netherlands
by Harwood Academic Publishers GmbH
Printed in Malaysia

### CASE REPORT

### Duodenal Varices: A Case Report and Review of the Literature

LAWRENCE McCHESNEY[1,*], DONALD JENSEN[2], TERENCE MATALON[3], DANIEL GANGER[2], HOWARD SANKARY[1], PRESTON FOSTER[1] and JAMES W. WILLIAMS[1]

[1]Transplantation Surgery, [2]Clninical Hepatology and [3]Invasive Radiology, Rush University Rush-Presbyterian-St. Luke's Medical Center, Chicago, Illinois 60612

KEY WORDS: Duodenal varices transjugular intrahepatic portosystemic shunt ectopic varices portocaval shunt extrahepatic portal hypertension.

### INTRODUCTION

Duodenal varices are a rare but frequently fatal cause of upper gastrointestinal bleeding. Although there are greater than 100 cases reported in the world literature, they still are rarely included in the differential diagnosis of gastrointestinal bleeding. A patient with bleeding duodenal varices is reported and the pertinent literature is reviewed.

### CASE REPORT

D.C. is a 56 year old male with a past medical history of excessive ethanol use and internal hemorrhoids. He presented in the summer of 1993 to another hospital with gastrointestinal bleeding secondary to an endoscopically confirmed gastric ulcer acquired following the withdrawal of alcohol use. He was placed on a regime of $H_2$ blockers, iron replacement therapy and did well for five months but then presented with complaints of progressive fatigue and epigastric discomfort. Evaluation revealed a hemoglobin of 3.8 g./dl. Colonoscopy did not reveal a source of bleeding. Esophagogastroduodenoscopy (EGD), revealed antral gastritis and duodenal varices and the absence of gastric or esophageal varices nor a duodenal ulceration. He was transferred to our institution for further evaluation. On presentation he was hemodynamically stable. His previous history in addition to his clinical evaluation and laboratory values all contributed to a diagnosis of cirrhosis with a Child's-Pugh Score of seven. His total bilirubin was 0.6 mg%, albumin 2.6 gm%, prothrombin time 12.1 seconds, the ammonia level was normal without encephalopathy, ascites or muscle wasting. His liver edge was 3–4 cm. below the right costal margin and his splenic tip was palpable. On his second hospital day he underwent a Transjugular Intrahepatic Portosystemic Shunt (TIPS) procedure. His portal vein pressure was 21 mm Hg with a portosystemic gradient of 10 mm Hg. A self-expandable Wallstent® was placed and his post stent gradient was 2mm Hg. Portography revealed the presence of esophageal, gastric and duodenal collaterals, with spleno-renal communications (Figure 1). His post procedural course was entirely uneventful and he was discharged the following day. He presented to the Emergency Ward three days later with melena, fatigue and a drop in his hemoglobin. EGD was repeated and no esophageal varices nor a gastric source of bleeding were identified. However, a large duodenal varix with a prominent erosion in the second portion of the duodenum was found with an associated blood clot (Figure 2). Ultrasonic evaluation of his TIPS showed

**Address for correspondence*: Section: Transplantation Surgery Rush-Presbyterian St. Luke's Medical Center.1653 W Congress Parkway Chicago, Illinois 60612.

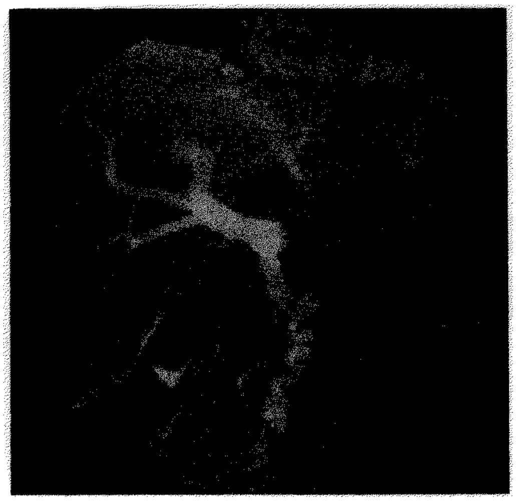

**Figure 1** Portography revealing the angiographic appearance of duodenal varices.

absence of flow through the shunt. Portography revealed mal-positioning of the stent; therefore, he underwent revision of his TIPS stent. Portal vein pressure was 13 mm Hg. with a portosystemic gradient of 4mm. Hg. Because of the history of previous early thrombosis of his TIPS, he underwent an ultrasonic examination of his shunt the day following revision of his stent. The repeat ultrasonic evaluation again failed to document flow through the intrahepatic shunt. With recurrent thrombosis of the TIPS, it was elected to proceed to a surgical porto-caval shunt. Celiotomy revealed micronodular cirrhosis which was confirmed by biopsy. During the initial intra operative course, an extrahepatic source of his portal hypertension was evident. Serosal varices of the duodenum were readily apparent. Upon transection of the portal vein, poor flow in the portal vein was identified. Passage of a balloon catheter in to the superior mesenteric vein resulted in the delivery of a large laminated clot. A normal quality of portal vein flow was then obtained. An end-to-side porta-caval anastomosis was then completed. His postoperative recovery was uneventful with absence of encephalopathy or hepatic insufficiency and he was discharged on the fifth post operative day. Repeat endoscopy two weeks later revealed the persistent presence of the duodenal varices but of a markedly reduced size and absence of the stigmata of recent bleeding.

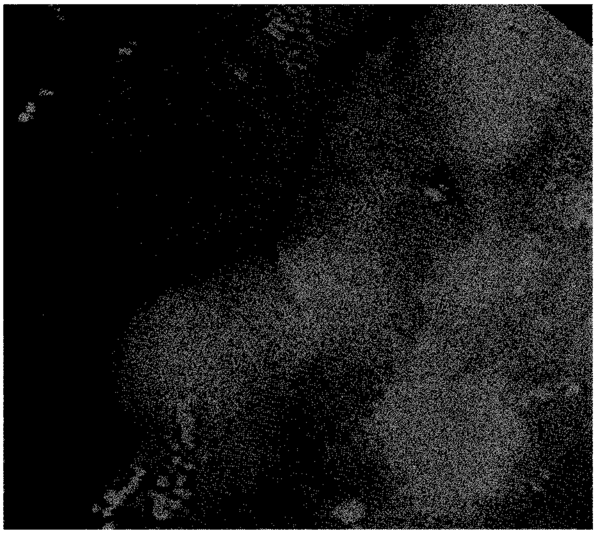

**Figure 2** Endoscopic manifestations of duodenal varices.

### DISCUSSION

Although duodenal varices are a rare source of gastrointestinal bleeding, they are associated with an abnormally high morbidity and mortality. In one series, a greater than 40% mortality rate has been reported after the treatment of duodenal varices[1]. We reviewed the literature to identify the salient features of this process.

*Alberti* has been credited with the first literature report on duodenal varices in 1931[2] applying the radiological findings described by *Wolf* for esophageal varices[3]. Since then, approximately 115 cases of duodenal varices have been reported in the world literature.

### *Pathophysiology*

Duodenal varices represent an ectopic portosystemic shunt[4]. Portosystemic communications in splanchnic hypertension occur through the following routes; i) by way of the gastroesophageal plexus to the azygous system, ii) via the hemorrhoid plexus, iii) via a recannulized umbilical vein and iv) via the pancreatoduodenal venous arcade to the retroperitoneal space to communicate with the inferior vena cava utilizing veins of Retzius[5]. Additionally, surgical or inflammatory adhesions of the intestines act as a route of portosystemic shunting[6]. Although duodenal varices as a source of gastrointestinal hemorrhage are rare, representing only one third of all ectopic sources of variceal bleeding, their angiographic prevalence is discordantly high. Paraduodenal varices were identified in 46 of 106 (40%), patients who underwent angiography for portal hypertension[7]. The anatomical reason proffered for this discrepancy is that duodenal varices occur on the serosal surface of the duodenum as well as in the muscular layers[8]. However, their clinical significance is not apparent until the varix expands into the submucosal space where it can produce hemorrhage into the gastrointestinal lumen. The location of the submucosal manifestation of duodenal varices was reviewed by *Amin et al.* and revealed that the duodenal bulb was the source of the varices in 55 of 73 cases.

Whether the presence of duodenal varices represents a manifestation of prolonged severe portal hypertension or merely a variation of portal hypertension is in debate. In one review 50% of the patients with duodenal varices had concomitant gastroesophageal varices[1]. An intrahepatic origin of portal hypertension was the most commonly identifiable anatomical source review by *Tanaka* of patients with duodenal varices[9]. However, in a review by *Amin et al.*, an extrahepatic etiology of portal hypertension *accounted for 39 of 56 (70%) reported cases.*[10] Again when *Stephan* and *Miething* examined the radiological findings of patients with duodenal varices, they found 40% had an extrahepatic source of portal hypertension[7]. This was confirmed by *Itzchak et al.* when duodenal varices were angiographically demonstrated in 18 of 20 cases of extrahepatc obstruction of the portal vein or obstruction of the splenic vein[11]. *Lebrec and Behamou* suggests that some form of ectopic varices occur in 20–30% of all extrahepatic origins of portal hypertension[6].

*Wheeler et al.*, submitted another etiology of duodenal varices when they reported a case of hepatic artery-portal vein fistula[12]. Additionally, duodenal varices may be the result of obstruction of the splenic or superior mesenteric veins. Here, the varices provide an alternate pathway around the obstruction connecting branches of the superior mesenteric or splenic vein upstream to reconstitute intrahepatic portal flow[13]. According to the review by *Khouqeer et al.*, duodenal varices are unlike colonic, and jejunal/ileal varices, in that they have not been associated with previous surgical adhesions[1]. *Eleftheriads* suggests yet another mechanism for the development of duodenal varices. He report the development of duodenal varices occurring after the loss of esophageal portosystemic shunts by ablative sclerotherapy[13].

### *Diagnosis*

Gastrointestinal bleeding as a result of duodenal variceal rupture presents as hematemesis, melena or both and are the only clinical manifestation of duodenal varices[6]. In fact, bleeding duodenal varices may well be confused with the findings of a bleeding duodenal ulcer. The fluoroscopic manifestations of duodenal varices on barium studies are those of irregular filling defects that might be confused with the presence of a duodenal tumor. It should be noted that many of the reported cases in the literature occurred prior to the liberal use of endoscopy. However, even with endoscopy, the accurate determination of duodenal varices is difficult. In one recent review, the correct preoperative endoscopic diagnosis was obtained in only 44% of the reviewed cases[1]. These errors occurred because the terminal portion of the duodenum was not visualized. Therefore, proper endoscopic evaluation must include all portions of the duodenum. Since duodenal varices may occur concomitantly with gastroesophageal varices, the identification of gastroesophageal varices does not rule out duodenal varices as a source of hemorrhage[14]. In reality, when the duodeum is filled with blood, a distinction between duodenal variceal versus a peptic or neoplastic etiology is seldom made. Therefore failure of direct endoscopic visualization of a discrete bleeding source should lead to selective angiography with venous phase imaging.

### *Management*

The suggested management of duodenal varices is just as varied as is its etiology. The first reported treatment was by *Wheeler* in 1957 in which his patient underwent suture ligation of the duodenal varices, followed by splenectomy with splenorenal anastomosis and subsequently internal, external, aneurysmorrhaphy and wiring[12].

The utilizing of systemic vasopressin for the control of duodenal variceal bleeding has not been shown to be reliable. However, improved results have been gained by selective infusion of vasopressin[15,16]. The use of routine balloon tamponade would be unsuccessful due to the distal site of the origin of the bleeding. Therefore, persistent bleeding despite a properly placed Sengstaken-Blakemore balloon should lead one to suspect a distal lesion in the presence of gastroesophageal varices.

Surgical methods utilized thus far included direct suture ligation of the varices via duodenotomy, and even duodenal resection. In one recent series, only 2 of 11 survived using this modality without the need for yet another definitive procedure to control bleeding[1]. In this same series, when ligation of the varices or resection of the duodenum failed to definitively control bleeding, these patients were further managed by the creation of a surgical portosystemic shunt. The choice of which shunt to utilize must be tailored to the etiology of the portal hypertension. To this extent, complete angiographic determination of the source of portal hypertension is required preoperatively.

Over the last few years, endoscopic sclerotherapy has proven useful in the management of bleeding duodenal varices[17,18,19,20,21]. To date there have been no reported cases of complications following duodenal sclerotherapy, but the potential for penetration and perforation of the thin duodenal wall is ever present. Additionally, no consensus has been reached regarding the benefits of prophylactic sclerotherapy for the long term management of duodenal varices[18]. Sclerotherapy does not result in the lowering of portal pressure and in fact it obliterates an established portosystemic collateral system as stated by *Eleftheriads*[3]. To support this theory, there have been three case reports of the development of duodenal varices occurring after successful sclerotherapy of esophageal varices[13,22]. There is also the theoretical risk that the sclerosant might gain access to the hepatic circulation analogous to the pulmonary vascular complications that occur after esophageal injections of sclerosing agents.

Other modalities for the management of duodenal varices include techniques utilizing invasive radiology. *Munu et al.* report a case where embolization of duodenal varices was achieved by the injection of isobutyl-cyanoacrylate[23]. Embolization using coils could afford yet another method[23]. However, recanalization of the obliterated veins or the development of additional collaterals could result in the need for subsequent medical or surgical therapy[18]. Another possibility, as utilized in our case, is the creation of a central portosystemic shunt via the Transjugular Intrahepatic Portosystemic Shunt. In the absence of an extrahepatic source of the portal hypertension, this modality should provide at least a temporizing effect. However, the patency rates after long term follow up of this technique may be low due to stenosis of the stent as a result of pseudointimal hyperplasia. The prevalence of pseudointimal hyperplasia stenosis has been reported to occur in 55–70% of stents in long term follow up[24,25].

### SUMMARY

Duodenal varices, although a rare source of gastrointestinal bleeding are associated with high morbidity and mortality. This may be a result of a delayed diagnosis. They may result from an intrahepatic origin of portal hypertension but have a high correlation with an extrahepatic origin. Their diagnosis is problematic and requires endoscopic visualization of the complete duodenum. Persistent bleeding despite control of an gastroesophageal source indicates the need for visceral angiography. Methods of management are varied and dependent on the identification of the anatomical source of portal hypertension. The creation of a portosystemic shunt either surgically or radiologically has been shown to result in control of bleeding. However, experience with endoscopic sclerotherapy is growing but its role requires wider evaluation.

### BIBLIOGRAPHY

1. Khouqeer, F., Morrow, C., Jordan, P. (1987) Duodenal varices as a cause of massive upper gastrointestinal bleeding. *Surgery*, **102**, 548–552.
2. Alberti, W. (1931) Uber den roentgenologixhen nachweis von varizen in buolbus duodeni, *Forsch Geb Rontgenstr.*, **43**, 60–65.
3. Wolf, G. (1928) Die Erkennug von osophagus varizen in rotgenbilde Fortsch Roentgenstr Nuklearmed Ergenzungs-band, **37**, 890–893.
4. Perchik, L., Max, T.C. (1963) Massive hemmorrhage from varices of the duodenal loop in a cirrhotic patient. *Radiology*, **80**, 47–49.
5. Schwartz, S., Shires, G., Spencer, F. (1989) Schwartz Principles and Practice of Surgery. Fifth Edition. Chapter **30**, 1356–1357. New York Cith, McGraw-Hill Book Co.
6. Lebrec, D., Behamou, J. (1985) Ectopic varices in portal hypertension. *Clin Gastroenterol.*, **14**, 105–119.
7. Stephen, G., Miething, R. (1968) Roentgendiagnostik varicoser duodenal-veranderungen bei portaler hypertension. *Radiologie*, **8**, 90.
8. Nardone, G., Budillon, G. (1991) Treatment of duodenal varices by endoscopic sclerotherapy. *Gastrointest Endosc.*, **37**, 407–408.
9. Tanaka, T., Kato, K., Taniguchi, T., Takagi, D., Takeyama, N. (1988) A case of ruptured duodenal varices and review of the literature. *Jpn J Surg.*, **18**, 595–600.
10. Amin, R., Alexix, R., Kowzis, J. (1985) Fatal ruptured duodenal Varis: A case report and review of literature. *Am. J Gastroenterol*, **80**, 13–18.
11. Itzchak, Y., Glickman, M. (1977) Duodenal varices in extrahepatic portal obstruction. *Radiology*, **124**, 619–24.
12. Wheeler, H.B., Warren, R. (1957) Duodenal varices due to portal hypertension from ateriovenous aneurysm. *Ann. Surg.*, **146**, 229–238.
13. Eleftheriads, E. (1988) Duodenal varices after sclerotherapy for esophageal varices. *Am. J Gastroenterol.*, **83**, 439–441.
14. Hellstrom, H., Hovat, B., McCoy, M. (1973) Duodenal varices with cirrhosis of the liver: report of three cases. *Am. Surg*, **39**, 518–521.
15. Kunert H, Ottejann R. (1976) Endoscopy in bleeding duodenal varices. *Endoscopy*, **8**, 99–101.
16. Salam, A., Goldman, M., Smith, D. Jr, Hill, H. (1972) Gastric, intestinal and gallbladder varices: hemodynamic and therapeutic considerations. *South Med*, **172**, 402–.
17. Barbish, A., Ehrinpreis, M. (1993) Successful endoscopic injection slcerotherapy of a bleeding duodenal varix. *Am J Gastroenterol*, **88**, 90–92.
18. Kirpatrick, J., Shoenut, J., Micflikier, A. (1985) Successful injection sclerotherapy for bleeding duodenal varix in intrahepatic portal obstruction. *Gastrointest Endosc*, **31**, 259–260.

19. Gertsch, P., Blumgart, L. (1988) Cure of bleeding duodenal varix by sclerotherapy. *Br J. Surg.*, **75**, 717.

20. Nardone, G., Budillon, G. (1991) Treatment of duodenal varices by endoscopic sclerotherapy. *Gastrointest Endosc.*, **37**, 407–408.

21. Herold, G., Stange, E. (1993) Sclerotherapy of duodenal varices using firbrin tissue sealant. *Endoscopy*, **25**, 371–372.

22. Sung, J., Chung, S., Leung, H., Lai, C., Ismael, A. Li, A. (1993) Duodenal varices in hepatocellular carcinoma. *Endoscopy*, **25**, 195.

23. Munu Y., Gayet, B., Nahum, H. (1987) Bleeding duodenal varices: Diagnosis and treatment by percutaneous portography and transcatheter embolization. *Gastrointest Radiol*, **12**, 111–113.

24. LaBerge, J., Ring, E., Gordon, R., Lake, J., Doherty, M., Somberg, K., Roberts, J., Ascher, N. (1993) Creation of transjungular intrahepatic portosystemic shunts with the wallstent enndoprosthesis; results in 100 patients. *Radiology*, **187**, 413–420.

25. Freedman, A., Sanyal, A., Tisnado, J., Cole, P., Shiffmann, M., Luketic, V., Purdum, P., Darcy, M., Posner, M. (1993) Complications of transjugular intrahepatic portosystemic shunt: A comprehensive review. *Radiographics*, **13**, 1185–1210.

